# Null Mutation of the *Fascin2* Gene by TALEN Leading to Progressive Hearing Loss and Retinal Degeneration in C57BL/6J Mice

**DOI:** 10.1534/g3.118.200405

**Published:** 2018-08-06

**Authors:** Xiang Liu, Mengmeng Zhao, Yi Xie, Ping Li, Oumei Wang, Bingxin Zhou, Linlin Yang, Yao Nie, Lin Cheng, Xicheng Song, Changzhu Jin, Fengchan Han

**Affiliations:** * Key Laboratory for Genetic Hearing Disorders in Shandong, Binzhou Medical, University, 346 Guanhai Road, Yantai 264003, Shandong, P. R. China; † Department of Human Anatomy and Histology and Embryology, Binzhou Medical University, 346 Guanhai Road, Yantai 264003, Shandong, P. R. China; ‡ Department of Biochemistry and Molecular Biology, Binzhou Medical, University, 346 Guanhai Road, Yantai 264003, Shandong, P. R. China; § Department of Otorhinolaryngology-Head and Neck Surgery, Yuhuangding Hospital, 20 East Yuhuangding Road, Yantai 264000, Shandong, P.R. China

**Keywords:** Fascin2, mutation, TALEN, hearing loss, hair cell, retinitis pigmentosa

## Abstract

Fascin2 (FSCN2) is an actin cross-linking protein that is mainly localized in retinas and in the stereocilia of hair cells. Earlier studies showed that a deletion mutation in human *FASCIN2* (*FSCN2*) gene could cause autosomal dominant retinitis pigmentosa. Recent studies have indicated that a missense mutation in mouse *Fscn2* gene (R109H) can contribute to the early onset of hearing loss in DBA/2J mice. To explore the function of the gene, *Fscn2* was knocked out using TALEN (transcription activator-like effector nucleases) on the C57BL/6J background. Four mouse strains with deletions of 1, 4, 5, and 41 nucleotides in the target region of *Fscn2* were developed. F1 heterozygous (*Fscn2^+/−^*) mice carrying the same deletion of 41 nucleotides were mated to generate the *Fscn2^−/−^* mice. As a result, the *Fscn2^−/−^* mice showed progressive hearing loss, as measured in the elevation of auditory brainstem-response thresholds. The hearing impairment began at age 3 weeks at high-stimulus frequencies and became most severe at age 24 weeks. Moreover, degeneration of hair cells and loss of stereocilia were remarkable in *Fscn2^−/−^* mice, as revealed by F-actin staining and scanning electron microscopy. Furthermore, compared to the controls, the *Fscn2^−/−^* mice displayed significantly lower electroretinogram amplitudes and thinner retinas at 8, 16, and 24 weeks. These results demonstrate that, in C57BL/6Jmice, *Fscn2* is essential for maintaining ear and eye function and that a null mutation of *Fscn2* leads to progressive hearing loss and retinal degeneration.

Age-related hearing loss (presbycusis), one of the most common perceptive diseases among the elderly population, causes both communication disorders and psychological problems(Gates and Mills 2005; Yamasoba *et al.* 2013; Yamasoba *et al.* 2007). In addition to environmental and social factors, genetic aspects are involved in pathogenesis in about 50–60% of people with age-related hearing loss(Han *et al.* 2015; Semaan *et al.* 2013).Developing effective methods to treat or prevent this condition is difficult due to the lack of molecular and cellular knowledge about the related disorders(Fujinami *et al.* 2012). Therefore, it is of great significance to study the mechanisms of genetic variance that lead to progressive hearing loss. As mice and humans share similar genetic components, anatomic structures, and pathological characteristics, mouse models play a crucial role in understanding the pathogenesis associated with these genes(Angeli *et al.* 2012; Noben-Trauth and Johnson 2009).

Fascins are actin-binding proteins that cross-link filamentous actin into tightly packed parallel bundles(Hashimoto *et al.* 2011). Fascin2 (fascin homolog 2, Fscn2; encoded by *Fscn2*) is first identified as a retina-specific transcript localizing in the inner and outer segments of bovine photoreceptor cells(Saishin *et al.* 2000; Saishin *et al.* 1997; Tubb *et al.* 2000). In humans, a naturally occurring frameshift mutation in *FSCN2*, 208delG, causes premature termination of protein translation. In Japanese patients, the 208delG heterozygosity correlated with autosomal-dominant retinitis pigmentosa and autosomal-dominant macular degeneration, with the mutation occurring in 3.3% of the cohort(Wada *et al.* 2003; Wada *et al.* 2001). Targeted disruption of *Fscn2* in mice, either by conventional exon-1 replacement or by knock-in of the 208delG point mutation, resulted in morphological alterations in retinal tissues, decreased electrical activity in rod and cone cells, and age-dependent photoreceptor degeneration(Yokokura *et al.* 2005). These experiments have indicated that the *FSCN2* gene plays an important role in the morphogenesis of photoreceptors.

Later studies have shown that mutations in *Fscn2* are related to hearing loss in DBA/2J mice. These mice develop early-onset hearing loss with auditory-evoked brainstem response (ABR) thresholds that are elevated by 15-20 dB at age 3 weeks and approach to deafness at about 3 months of age(Yang *et al.* 2015; Zheng *et al.* 1999). Apart from an age-related hearing locus (also known as *ahl)* in *cdh23*, a locus named *ahl8* recently has been identified as the main contributor to early-onset hearing loss in DBA/2J mice(Johnson *et al.* 2008). The *ahl8*-causative gene has been identified as *Fscn2*; it encodes an actin cross-linking protein that was previously thought to be retina-specific(Shin *et al.* 2010). Further studies show that the *Fascin2* p.R109H mutant binds but fails to efficiently cross-link the actin filaments, which indicates that Fscn2 functions to slow actin depolymerization at the tips of the stereocilia to maintain their length(Perrin *et al.* 2013). In mice or chickens, Fscn2 is abundant in hair-cell stereocilia and is colocalized with F-actin(Shin *et al.* 2010). It is a monomeric globular protein with two actin binding sites that may stabilize stereocilia by means of cross-linking adjacent filaments(Jansen *et al.* 2011). Actually, Fscn2 can bind with multiple actins, including ACTA1, ACTA2, ACTB, ACTC1, ACTG1, and ACTG2(Avenarius *et al.* 2014). Fscn2 has also been reported to bind with β-, γ-, and ɑ-actin with similar affinity(Perrin *et al.* 2013; Shin *et al.* 2010).

Although there are reports on mice with targeted disruptions of *Fscn2(**Yokokura et al. 2005**)*, the hearing loss phenotype has not been reported in these animals. It is interesting that, apart from progressive hearing loss starting at age 3 weeks, the DBA/2J mice between 3 and 11 months old also undergo age-related retinal degeneration(Schuettauf *et al.* 2004), which may be caused by *ahl* and/or *ahl8* alleles. To clarify the function of *Fscn2*, a null mutation was induced in the gene in C57BL/6J mice using TALEN (transcription activator-like effector nucleases) techniques(Sung *et al.* 2013). The results revealed that the *Fscn2* gene plays an essential role in mouse ears and eyes; mice with the null mutation of *Fscn2* develop progressive hearing loss and retinal degeneration.

## Materials and Methods

### Gene targeting and generation of *Fscn2*^−/−^ mice

Experimental mice were bred in a specific pathogen-free animal facility at Binzhou Medical University. The animal studies were conducted in accordance with the principles set forth in the Guide for the Care and Use of Laboratory Animals of Binzhou Medical University and were approved by that university’s Institutional Animal Use and Care Committee (protocol 14-0514). A total of 354 littermates with ages from 3 to 52 weeks were included in this study, with 185 *Fscn2^−/−^* mice in the experimental groups and 169 wild-type mice in the control groups.

### Genotyping for mouse identification

The wild-type mice (*Fscn2*^+/+^), heterozygous mice (*Fscn2*^+/−^), and homozygous mice (*Fscn2*^−/−^) were genotyped using a polymerase chain reaction (PCR). The primers (Fscn2-KF and Fscn2-KR; listed in Table 1) for genotyping were designed to span the mutation region; they were synthesized by Sangon Biotech Co. Ltd. (Shanghai, China). Mouse genomic DNA was extracted from tail tips for PCR analysis, as described(Han *et al.* 2012). DNA concentration was measured using NANO DROP 2000C (Thermo Scientific, MA, USA). The PCR mixture contained 12.5 µL of the 2×EasyTaq PCR SuperMix (TRANS, Beijing, China), 200 ng DNA, and 1 µL of each 10 µM primer; ddH_2_O was added to make a total mixture of 25 µL. The reaction conditions were as follows: denaturation at 94° for 3 min; 30 cycles of 94° for 30 sec, 64° for 40 sec, and 72° for 50 sec; and a final period at 72° for 5 min. The PCR products were identified by use of agarose gel electrophoresis. The images were recorded using the FluorChem HD2 System (ProteinSimple, CA, USA).

**Table 1:** Primers for genotyping and RT-PCR

ID	Sequence	Product Size (bp)
Gapdh-F	5′-CTTCCGTGTTCCTACCCCCAATGT-3′	100
Gapdh-R	5′-GCCTGCTTCACCACCTTCTTGATG-3′	
Fscn2-F1	5′-CCAGGTGCTGAAGATCCAGT-3′	879
Fscn2-R1	5′-GTCTCCTGGTCGATTTGCAT-3′	
Fscn2-F2	5′-GCTTTGGCTTCAAGGTCAAC-3′	849
Fscn2-R2	5′-CCCCAGTGCTGGAATAGAAA-3′	
Fscn2-KF	5′-ATTGGAGCAGGTAGCGTCCATGTC-3′	394
Fscn2-KR	5′-TCACAGGCCACACGTCCATCTTC-3′	

### Assays for gene transcription

Transcription of *Fscn2* was evaluated using reverse-transcription polymerase chain reaction (RT-PCR)(Arno *et al.* 2016; Han *et al.* 2015; Johnson *et al.* 2008). The total RNA in the inner ears and eyes of *Fscn2^+/+^* and *Fscn2^−/−^* mice were extracted using TRIzol reagent (Invitrogen, Carlsbad, CA, USA), and cDNA was synthesized using Random Hexamer primers following the First Strand cDNA Synthesis Protocol (Roche Diagnostics, Indianapolis, IN, USA). Two sets of primers (Fscn2-F1 and Fscn2-R1; Fscn2-F2 and Fscn2-R2; Table 1) were designed for the PCR. The mixture of PCR contained 10 µL of the master mix, 1 µL of cDNA, and 1 µL of each of the 10 µM primers; 7 µL of ddH_2_O was added to make the total volume 20 µL. The thermal cycles included denaturation at 95° for 2 min; 30 cycles of 94° for 30 sec, 60° for 40 sec, and 72° for 50 sec; and a final period at 72° for 2 min. The PCR products were identified by agarose gel electrophoresis. The PCR products using primers (Fscn2-F1 and Fscn2-R1) spanning the deletion were finally sequenced. GAPDH was used as the internal control.

### Histological analyses of retina

Paraffin sections were made following the previous methods(Arno *et al.* 2016; Tian *et al.* 2010; Yan *et al.* 2017). The mice (5 in each group) were anesthetized with 2% tribromoethanol and then decollated. The eye tissues were isolated and fixed in 4% paraformaldehyde in 0.1 M phosphate-buffered saline (PBS; 7.4 pH) at 4° for 24 hr. The tissues were embedded in paraffin and serially sectioned in 5 μm thickness. The sections were mounted on glass slides and counterstained in hematoxylin and eosin. The retina was observed using light microscopy (Leica DMI4000 B, Leica Microsystems, Wetzlar, Germany). Histological analysis of retina was carried out following the previously described procedures(Obin *et al.* 2000). The following measurements were made for mice aged 4, 8, 16 and 24 weeks. (1) outer nuclear layer (ONL) cell density, assessed as the number of nuclei between the outer plexiform layer (OPL) and the photoreceptor inner segments (IS) along a 25 µm width transect (2) ONL thickness (distance from OPL to IS); (3) inner segments (IS) and outer segments (OS) thickness (distance from the ONL to the choroid membrane of the retina.

### Immunofluorescence observation of Fscn2 expression in retinas

Paraffin sections were made as described above. The sections were immersed in 0.5% Triton X-100 for 30 min and blocked with 5% bovine serum albumin for 60 min at room temperature. Primary antibodies (ab78599 goat anti-*Fascin2*, 1:400, Abcam) were diluted in 0.01 M PBS. The samples were incubated overnight at 4° with primary antibodies. After being washed three times with 0.01 M PBS, the samples were incubated with secondary antibodies (ab150145 rabbit anti-goat Alexa-488, 1:500, Abcam) at room temperature for 1 hr and then diluted in the 0.01 M PBS. Counterstaining was completed using Hoechst33342 (3 min at room temperature). Finally, the samples were mounted with VECTASHIELD Mounting Medium H-100 (Vector Laboratories, CA, USA) and were observed under immunofluorescent confocal microscopy (LeicaDMI4000 B, Leica Microsystems, Wetzlar, Germany).

### Western blotting

Western blotting was carried out following the previously described procedures(Arno *et al.* 2016; Cai *et al.* 2017; Han *et al.* 2015). Briefly, *Fscn2^+/+^* and *Fscn2^−/−^* littermate mice were decollated, and their cochleae were dissected. The cochlear proteins were extracted, and 30 µg of samples were separated using sodium dodecyl sulfate polyacrylamide gel electrophoresis and transferred to a polyvinylidene fluoride membrane (Millipore, USA). The membrane was immersed in 5% nonfat milk and buffered in Tris-buffered saline containing 0.1% Tween-20 at room temperature for 1.5 hr; it was then incubated at 4° overnight with primary antibody (ab111601, rabbit anti-*Fascin2*, 1:3000, Abcam) and β-actin (YT0099, rabbit anti-β-actin 1:2000, ImmunoWay). After being washed three times with the Tris-buffered saline, the membrane was incubated with horseradish peroxidase and conjugated with goat anti-rabbit secondary antibody (ab97051, 1:5000 dilution, Abcam) at room temperature for 1 hr. Protein expression was detected using SuperSignal West Pico (Thermo Scientific, MA, USA) and was photographed in a chemiluminescence instrument (3100Mini, Clinx Science, Shanghai, China).

### Measurement of ABR thresholds and DPOAE (distortion product otoacoustic emission) amplitudes

The mice were anesthetized with 2% tribromoethanol (0.2 mL per 10 g of body weight) and then placed on a heating pad to maintain a temperature of 37°. All operations were carried out in a soundproof and electromagnetic shielding room. We used the IHSS Smart EP 3.30 and USBez Software (Intelligent Hearing Systems) to measure the ABR thresholds and the DPOAE amplitudes at various intervals (3, 4, 5, 6, 8, 10, 16, 20, 24, 32, 40, and 52 weeks of age) in *Fscn2^−/−^* mice and *Fscn2^+/+^* mice (between 6 and 10 for each group at each of the corresponding time points), following the methods described previously(Han *et al.* 2012; Han *et al.* 2013).

### Electroretinogram (ERG) recording

The ERGs were recorded in *Fscn2^+/+^* mice and *Fscn2^−/−^* mice (between 6 and 8 in each group for each of the corresponding time points) with reference to the methods described previously(Arno *et al.* 2016; Yan *et al.* 2017; Yokokura *et al.* 2005). Briefly, the mice were dark-adapted (scotopic) overnight and were then prepared for the recordings in dim red light. The mice were anesthetized with 2% tribromoethanol and placed on a heating pad to maintain a temperature of 37°.The pupils were dilated with 0.1% phenylephrine HCl. Needle electrodes were placed just under the skin, with the ground electrode placed at the root of the tail and the reference electrode underneath the right corner of the mouth. The ring-shaped (active) electrode was placed close to the right eye’s cornea. A visual electrophysiology examining system (ROLAND RETI-port/scan 21, ROLAND CONSULT Stasche Finger GmbH, Germany) was used to record the ERG. The signals were amplified 10,000 times, and the band-pass was filtered at 0.1 to 1000 Hz.

### Observation of hair cells in the cochleae

The experiments were carried out following the method described(Han *et al.* 2012; Han *et al.* 2015; Yang *et al.* 2015). The mice in the *Fscn2^+/+^* and *Fscn2^−/−^* groups were observed at ages 3, 5, 8, 10, 16, 20, 24, 32, 40, and 52 weeks (n = 6 in each group), with a focus on their hair cells and stereocilia. After fixation and decalcification, the organs of Corti were carefully microdissected out and subdivided into the base, middle, and apex turns. Then, the tissues were mounted with glycerin on glass slides. The surface preparations were stained for F-actin with Alexa Fluor-488 conjugated with phalloidin (1:500 dilution, Invitrogen, CA, USA) for 1 hr at room temperature to show the hair cell bundles. Then, the samples were mounted with VECTASHIELD Mounting Medium H-100 (Vector Laboratories, CA, USA). After being washed three times in 0.01 M PBS (pH 7.4), the samples were examined with a fluorescent microscope (LeicaDM4000B, Leica Microsystems, Wetzlar, Germany). In each turn of cochlea, counts of hair cells in three continuous views were recorded. Hair cells were counted as present if the hair cell bundles’ V shapes were intact. The percentages of hair cells missing in the three turns of the organs of Corti were calculated and analyzed.

### Scanning electron microscopy

Scanning electron microscopy was carried out following the methods described previously(Arno *et al.* 2016). Briefly, the inner ears of the mice from each group (n = 5) were dissected outside of the skull, fixed in 2.5% glutaraldehyde phosphate buffer (0.1 M PBS) at 4° overnight, decalcified, and then washed 3 times in 0.1 M PBS (10 min each time). The organs of Corti were exposed after the overlying bones and membranes had been carefully cut off. The mouse cochleae were processed in 1% osmium tetroxide acid (postfixation) for 40 min, dehydrated with gradient alcohol (50%, 70%, 80%, 95%, and 100%), and dried to a critical point with liquid CO_2_. The samples were then mounted onto round nails made of pure copper and sputter-coated to produce a gold coat of 10-15 nm; finally, the samples were examined at 10 kV with an EVO MA 15/LS scanning electron microscope (Carl Zeiss, Oberkochen Germany).

### Statistical analysis

The analysis of variance test was used to analyze the data for the ABR thresholds, DPOAE amplitudes, and ERG amplitudes. Data regarding hair-cell loss were analyzed using the χ^2^ test. *P* < 0.05 was considered to be significant. Error bars represent the standard error of the mean.

### Data availability

The authors affirm that all data necessary for confirming the conclusions of this article are represented fully within the article and its tables and figures.

## Results

### Gene targeting and generation of *Fscn2^−/−^* mice

**Figure 1:**
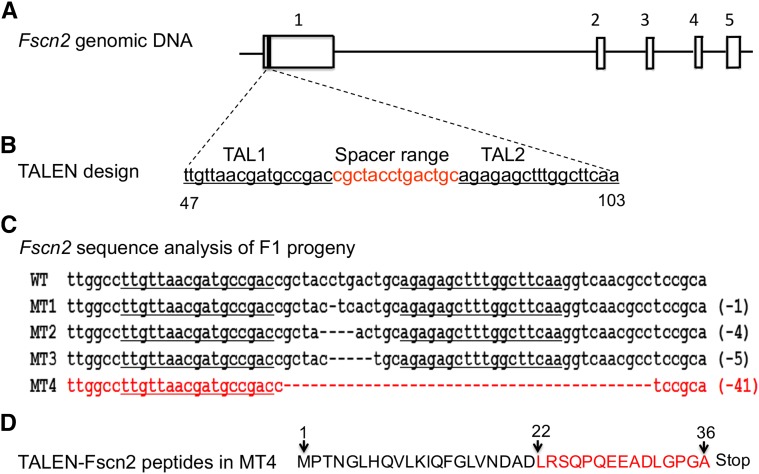
*Fscn2* gene knocked out in C57BL/6J mice using transcription activator-like effector nucleases. (A) *Fscn2* genomic DNA. The *Fscn2* gene contains 5 exons, which are indicated by the numbers 1 through 5. (B), The transcription activator-like effector nuclease (TALEN) targeting region in *Fscn2*; this region is at the first exon of *Fscn2*, including the sequence of TAL-L (TTGTTAACGATGCCGAC), the spacer range (cgctacctgactgc), and the sequence of AL-1-R (TTGAAGCCAAAGCTCTCT). (C) Sequence analysis of the *Fscn2* targeting region in the F1 progeny. The sequence from the wild-type mouse is labeled WT. Those from mutant types of strains are labeled MT1, MT2, MT3, and MT4; each includes a deletion of nucleotides. The sequence in MT4 that contains a deletion of 41 bp of nucleotides is highlighted in red. (D) The deduced amino acid sequence of *Fscn2* in MT4 mice. TALEN mutation in *Fscn2* produces a peptide with 36 amino acid residuals; 15 of these (highlighted in red) come from frame-shift alterations.

The *Fscn2* knockout mice were generated on the C57BL/6J background from Cyagen Biosciences (Guangzhou, China). The *Fscn2* null mutation was made using the TALEN technique, with two vectors targeting the first exon of *Fscn2* (Figure 1). The targeting region included sequences of the TAL-L (TTGTTAACGATGCCGAC), the spacer range (cgctacctgactgc), and the TAL-1-R (TTGAAGCCAAAGCTCTCT). The TALEN mRNA was then injected into the cytoplasm of the one-cell embryos(Wefers *et al.* 2013). The endonuclease generated DNA double-strand breaks at the spacer range; these breaks could be repaired by nonhomologous end joining(Sung *et al.* 2013; Wefers *et al.* 2013). Four mutant founders (F0) were generated, carrying the deletion of 1, 4, 5, and 41 nucleotides, respectively, with the 5′-end within the spacer range of *Fscn2*. Each mutation was predicted to alter the open reading frame of *Fscn2* and lead to the production of a truncated and function-null peptide. The F0 strains were crossed with C57BL/6J mice to generate the F1 strains. Male and female F1 heterozygous (*Fscn2^+/−^*) mice carrying the same deletion of 41 nucleotides were mated to generate the *Fscn2^−/−^* mice, which were maintained for 12 generations.

**Figure 2:**
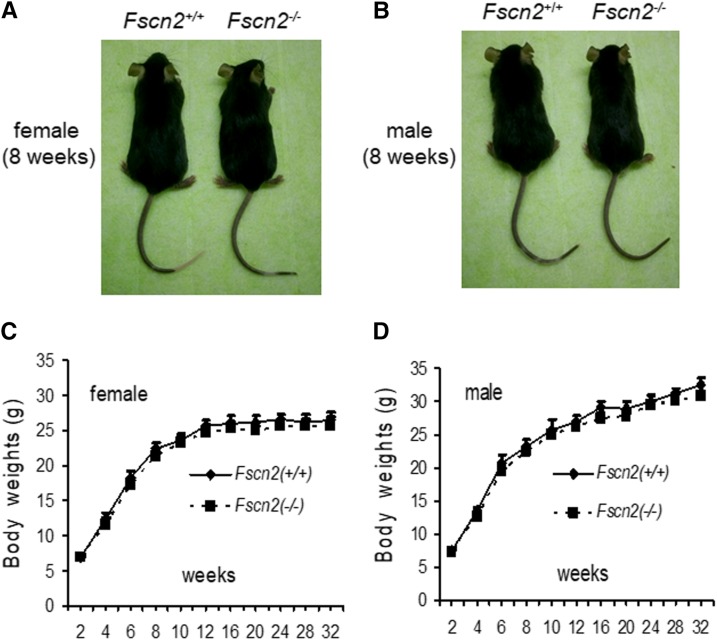
The shapes and weights of *Fscn2^−/−^* mice. The female (A) or male (B) *Fscn2^−/−^* mice showed nearly identical body shapes to those of the female or male wild-type mice. There is no significant difference for the weights of female or male mice between the two mouse groups in the observed period (C, D).

Overall, the mutation doesn’t influence the reproductivity of the mice. The number of pups ranged from 6-9 per litter for the *Fscn2^−/−^* mice and the wild-type mice. The weights of *Fscn2^−/−^* mice and wild-type mice are shown in Figure 2. There is no significant difference for the weights of female or male mice between the two mouse groups in the observed period.

### Genotyping and identification of *Fscn2^−/−^* mice

**Figure 3:**
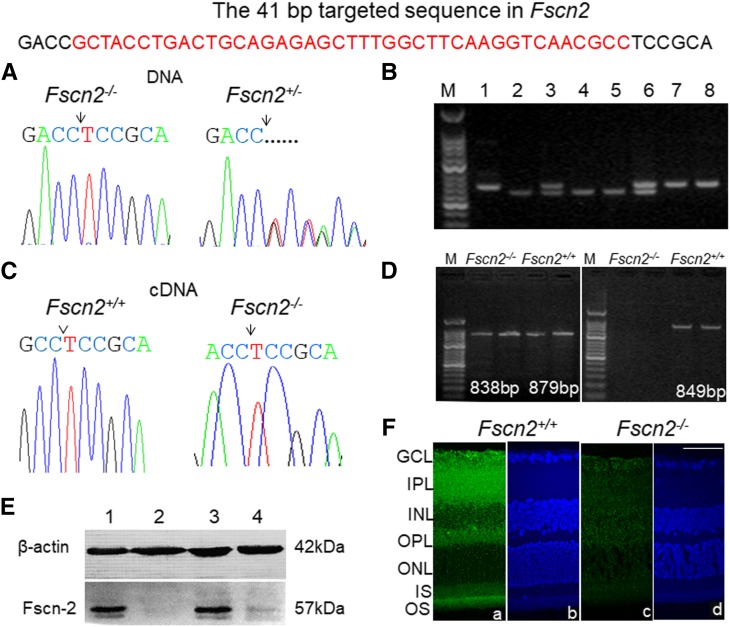
Identification of the *Fscn2* knockout mice. (A) DNA sequence spanning the deletion region in the homozygous mouse *Fscn2^−/−^* and the heterozygous mouse (*Fscn2*^+/−^). The deletions are indicated by an arrow or by a dotted line. (B) Agarose gel electrophoresis of the polymerase chain reaction (PCR) products for genotyping of the mouse strains. Lane M shows 50 bp DNA ladders; lanes 1, 7, and 8 show PCR products from *Fscn2^+/+^* mice; lanes 3 and 6 show products from heterozygous *Fscn2^+/−^* mice; lanes 2, 4, and 5 are those from *Fscn2^−/−^* mice. (C) The cDNA sequence spanning the deletion region in *Fscn2^−/−^* mice. The cDNA sequence downstream of the targeting position from *Fscn2^+/+^* mice is indicated by a caret. The position of deletion in *Fscn2^−/−^* mice is indicated by an arrow. (D) The mutant identification (using RT-PCR) in the inner ears. The left image shows RT-PCR products using primers that span the mutation area and the first intron between exon 1 and exon 2. The PCR products, which are of similar sizes (only 41 bp different), are amplified from the cDNA of both strains. The right image shows the PCR products using a forward primer within the targeted region. RT-PCR products from *Fscn2^−/−^* mice can not be amplified. (E) Fscn2 expression in the cochleae using Western blotting. *Fscn2^+/+^* and *Fscn2*^+/−^ mice express Fscn2 protein in the cochleae, but *Fscn2^−/−^* mice show no Fscn2 expression. (F) Expression and immunolocalization of Fscn2 in retinas of *Fscn2^+/+^* and *Fscn2^−/−^* mice. Fscn2 is expressed mainly in inner plexiform layers (IPL), outer plexiform layers (OPL), and outer segments (OS) of *Fscn2^+/+^* mouse retinas (panel 1); the protein is lost in *Fscn2^−/−^* mouse retinas (panel 3). The retinas counterstained by Hoechst33342, from *Fscn2^+/+^* (panel 2) and *Fscn2^−/−^* (panel 4) mice, are used as internal controls. GCL, ganglion cell layer; INL, inner nuclear layer; ONL, outer nuclear layer; IS, inner segment. Scale bars = 50 µm.

The Sanger sequencing results confirmed that the wild-type mice carried the *Fscn2* alleles (41 bp) and that the heterozygous *Fscn2^+/−^* mice and homozygous *Fscn2^−/−^* mice carried the mutation in one allele and both alleles, respectively (Figure 3A). A genotyping method for *Fscn2* was then developed to identify the wild-type mice (*Fscn2^+/+^*), heterozygous mice (*Fscn2^+/−^*), and homozygous mice (*Fscn2^−/−^*; Figure 3B). The deletion was also identified by RT-PCR (Figure 3D) and confirmed by sequencing the *Fscn2* cDNA from the inner ears (or eyes) of the wild-type mice and homozygous mice (Figure 3C). The deletion was supposed to produce a truncated peptide with 36 amino acid residuals. Western blotting results revealed that the native *Fascin2* protein was not expressed in the cochleae of the homozygous mice (*Fscn2^−/−^*), unlike in the wild-type or heterozygous mice (Figure 3E). Immunohistochemical staining showed that Fscn2 was mainly expressed in inner plexiform layers (IPLs), outer plexiform layers (OPLs), and outer segments (OSs) of the retinas in *Fscn2^+/+^* mice. However, Fscn2 was not expressed in any layer of the *Fscn2^−/−^* mouse retinas (Figure 3F).

### Early onset of progressive hearing loss in *Fscn2^−/−^* mice

**Figure 4:**
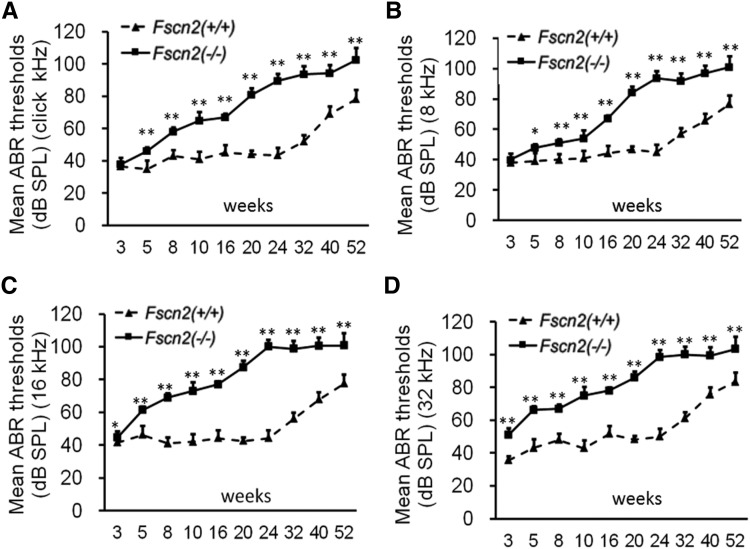
Observation of the auditory-evoked brainstem response thresholds in *Fscn2^−/−^* mice. Auditory-evoked brainstem response (ABR) thresholds tested in *Fscn2^−/−^* and *Fscn2^+/+^* mice at 10 time points from 3 to 52 weeks of age. (A) Measured at click stimulus frequency. (B) Measured at 8 kHz stimulus frequency. (C) Measured at 16 kHz stimulus frequency. (D) Measured at 32 kHz stimulus frequency. There were 7 to 10 mice in each *Fscn2^−/−^* group and 6 to 10 mice in each *Fscn2^+/+^* group at each time point. Error bars represent the standard error of the mean. * P < 0.05; ** *P* < 0.01

To observe the effects that *Fscn2* gene have on mouse hearing, ABR thresholds were measured in the *Fscn2^−/−^* and *Fscn2^+/+^* mice in a time-course manner. Generally, the ABR thresholds in the *Fscn2^−/−^* mice increased with age from 3 to 24 weeks; they reached a plateau of about 100 dB SPL at later time points (32, 40, and 52 weeks of age) at all stimulus frequencies (click and 8, 16, and 32 kHz). However, the ABR thresholds in the *Fscn2^+/+^* mice did not change until 24 weeks of age. Unlike those of the *Fscn2^+/+^* mice, the ABR thresholds in the *Fscn2^−/−^* mice had already significantly risen at stimulus frequencies of 16 and 32 kHz by age 3 weeks, which indicated an early onset of hearing impairment for high frequencies. Later on, at all time points and all stimulus frequencies, the ABR thresholds in *Fscn2^−/−^* mice were significantly higher than the levels for wild-type mice (Figure 4). The results in the *Fscn2^−/−^* mice demonstrated progressive hearing loss starting at age 3 weeks, leading to near deafness at age 24 weeks.

### Functional impairment of outer hair cells in *Fscn2^−/−^* mice

**Figure 5:**
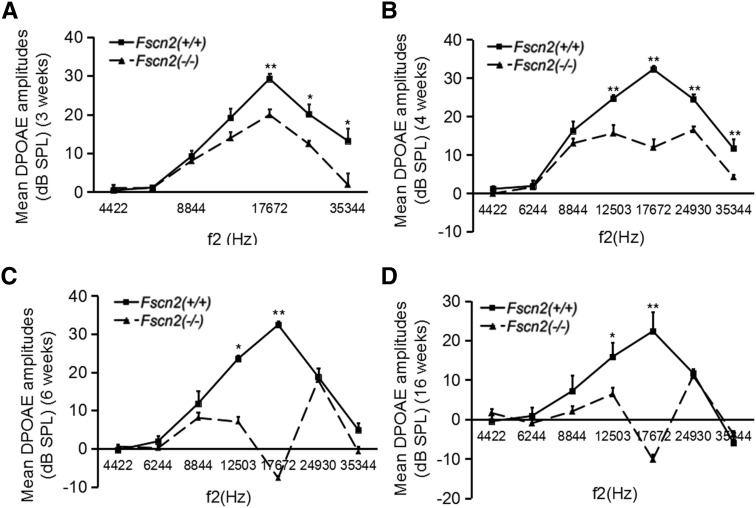
Observation of the distortion product otoacoustic emission amplitudes in *Fscn2^−/−^* mice. The distortion product otoacoustic emission (DPOAE) amplitudes measured in *Fscn2^−/−^* and *Fscn2^+/+^* mouse groups at f2 frequencies in the range of 4422 to 35 344 Hz. (A) Measured at 3 weeks. (B) Measured at 4 weeks. (C) Measured at 6 weeks. (D) Measured at 16 weeks. There were 7 to 10 mice in each *Fscn2^−/−^* mouse group and 6 to 10 in each *Fscn2^+/+^* group at each time point. * *P* < 0.05; ** *P* < 0.01

To evaluate the function of outer hair cells (OHCs), the amplitudes of DPOAE were measured in *Fscn2^−/−^* mice at ages 3, 4, 6, and 16 weeks at f2 frequencies from 4422 to 35 344 Hz. Typically, DPOAE amplitudes in *Fscn2^+/+^* littermates in the age range 3 to 16 weeks showed an inverted V curve, with the highs corresponding to an f2 frequency of 17 672 Hz. However, the DPOAE amplitudes in *Fscn2^−/−^* mice at age 3 weeks were lower than those of the *Fscn2^+/+^* mice at f2 frequencies from 8844 to 35 344 Hz—especially at 17 672 Hz. DPOAE amplitudes declined rapidly in *Fscn2^−/−^* mice and became negative at age 6 weeks (or older) at f2 frequency of 17 672 Hz (Figure 5). These results indicated an early progressive functional impairment of OHCs in *Fscn2^−/−^* mice.

### Progressive degeneration of hair cells in the cochleae of *Fscn2^−/−^* mice

**Figure 6:**
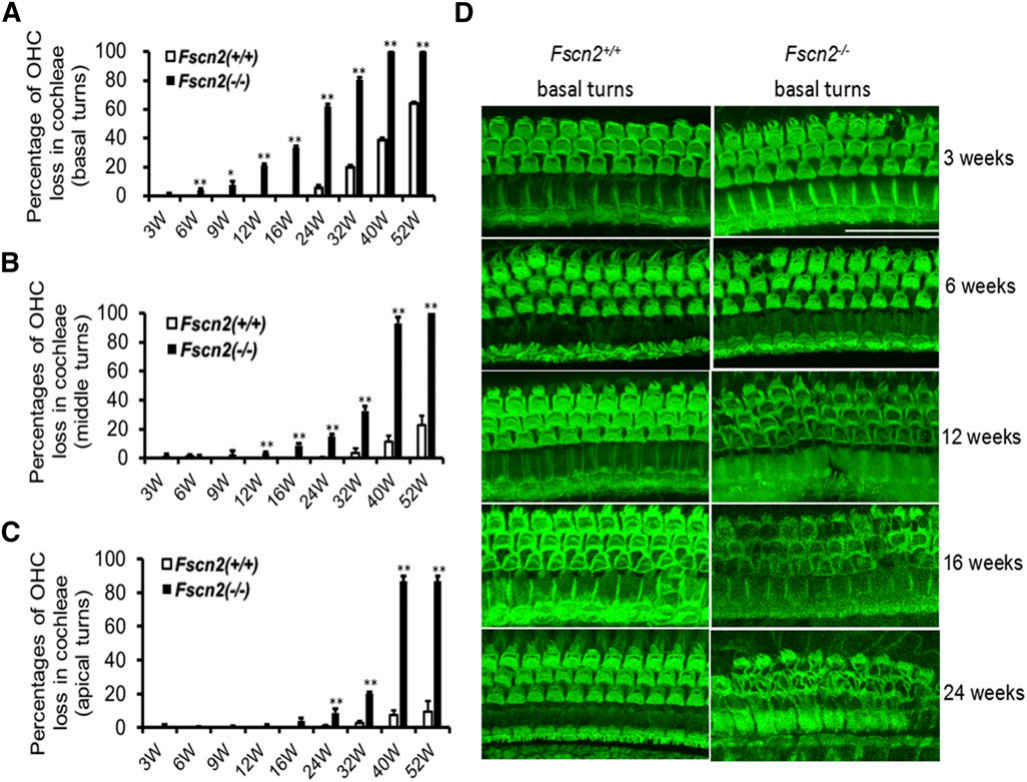
Loss of outer hair cells in the cochleae of *Fscn2^−/−^* mice. Hair cells were stained for F-actin with Alexa Fluor-488-labeled phalloidin. Overall, outer hair cell (OHC) loss in the cochleae of *Fscn2^−/−^* and *Fscn2^+/+^* was progressive. (A) OHC loss beginning in the basal turns. (B) OHC loss spreading to the middle turns. (C) OHC loss spreading to the apical turns. The percentages of OHC loss for *Fscn2^−/−^* mice were significantly higher than those for *Fscn2^+/+^* mice by age 6 weeks in the basal turns, by age 12 weeks in middle turns, and by age 24 weeks in the apical turns (n = 6 for each group; *P < 0.05; **P < 0.01). (D) Representative hair-cell images in the basal turns of *Fscn2^−/−^* and *Fscn2^+/+^* mice at ages 3, 6, 12, 16, and 24 weeks. The OHC images were normal and intact in *Fscn2^+/+^* mice, but in *Fscn2^−/−^* mice, loss of OHC occurred at age 3 weeks and became severe at older ages, especially by ages 16 and 24 weeks. Scale bar = 50 µm.

Hair cells were stained for F-actin with Alexa Fluor-488-labeled phalloidin. Degeneration of hair cells in the cochleae of *Fscn2^−/−^* mice was observed at various ages (3, 6, 9, 12, 16, 24, 32, 40, and 52 weeks). OHCs were counted as present if the V shapes of the hair bundles were intact. The hair-cell loss occurred in the basal turns of the cochleae as early as 6 weeks of age (3–4%); it spread to the middle and apical turns at ages 9 and 16 weeks, respectively. More than 80% of OHCs were missing in the basal turns by age 32 weeks, whereas the same number of OHCs was lost in the middle and apical turns at age 40 weeks (Figure 6A-6C). Representative hair-cell images of the basal turns in the *Fscn2^−/−^* and *Fscn2^+/+^* mice are shown in Figure 6D. The OHCs in *Fscn2^+/+^* mice were normal and intact in the age range 3 to 24 weeks, whereas in *Fscn2^−/−^* mice, the loss of OHCs occurred at age 6 weeks and became severe starting at 12 weeks.

**Figure 7:**
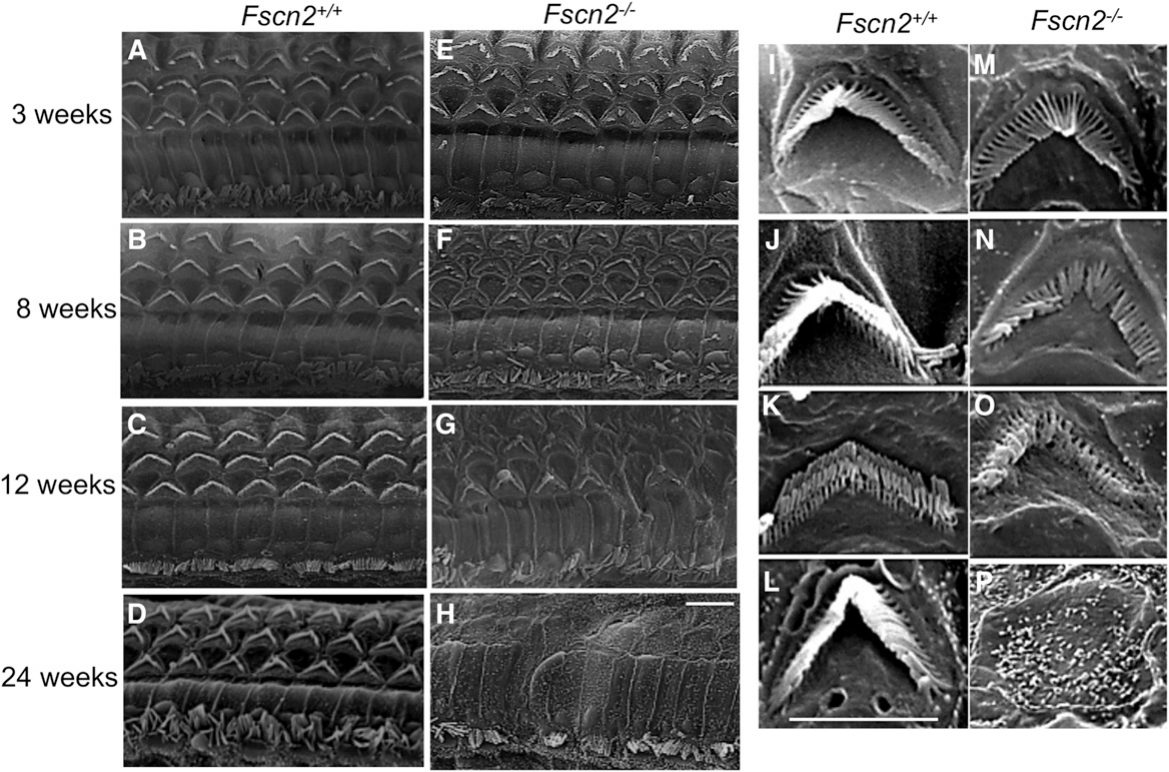
Alterations of stereocilia in the basal turns of cochleae in *Fscn2^−/−^* mice. Alterations of stereocilia of *Fscn2^−/−^* mice observed using scanning electron microscopy. *Fscn2^+/+^* mice showed normal appearance of stereocilia in OHCs in the basal turns of cochleae. (A) Image taken at age 3 weeks. (B) Image taken at age 8 weeks. (C) Image taken at age 12 weeks. (D) Image taken at age 24 weeks. However, stereocilia in the *Fscn2^−/−^* mice were abnormal. (E) Irregular stereocilia at age 3 weeks. (F) Disrupted stereocilia at age 8 weeks. (G) Disrupted stereocilia at age 12 weeks. (H) Completely lost stereocilia at age 24 weeks. Impairment in stereocilia of the inner hair cells was also remarkable in *Fscn2^−/−^* mice (E-H). The bundles of stereocilia in *Fscn2^+/+^* mice showed normal morphology at ages 3, 8, 12, and 24 weeks (i-I). However, those of stereocilia in *Fscn2^−/−^* mice were shorter in lengths and weaker in stiffness at age 3 weeks (M), became collapse at ages 8 and 12 weeks(N, O) and were completely lost at age 24 weeks (P), compared with those in *Fscn2^+/+^* mice. Scale bars = 5 μm.

Meanwhile, in *Fscn2^−/−^* mice, alterations of the stereocilia in the cochlear basal turns were observed using scanning electron microscopy (Figure 7). The images revealed that the stereocilia in the *Fscn2^−/−^* mice were already irregular at age 3 weeks, were disrupted at ages 8 and 12 weeks, and were completely lost at age 24 weeks. Compared with the bundles of stereocilia in *Fscn2^+/+^* mice, those in *Fscn2^−/−^* mice were much shorter and much less stiff at ages 8 and 12 weeks. Impairment of the stereocilia in the inner hair cells was also remarkable in *Fscn2^−/−^* mice.

### Progressive vision impairment and retinal degeneration in *Fscn2^−/−^* mice

**Figure 8:**
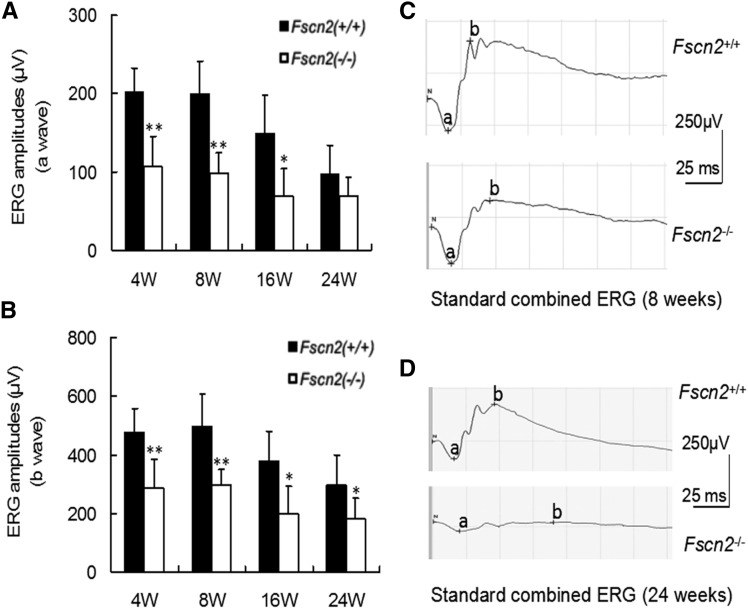
Dark-adapted flash electroretinogram of *Fscn2^−/−^* mice at age of 4, 8, 16, and 24 weeks. (A) The a-wave amplitudes of standard combined electroretinogram (ERG) reduced in *Fscn2^−/−^* mice compared with those of the *Fscn2^+/+^* mice at all time points. (B) The b-wave amplitudes of standard combined ERG reduced in *Fscn2^−/−^* mice and *Fscn2^+/+^* mice at all time points. (C) Typical a-waves of standard combined ERG at age 8 weeks. (D) Typical b-waves of standard combined ERG at age 24 weeks. The stimulus intensity of all recordings was 1.0 cd/m^2^, and n = 6 at each time point for each group. * *P* < 0.05; ** *P* < 0.01

**Figure 9:**
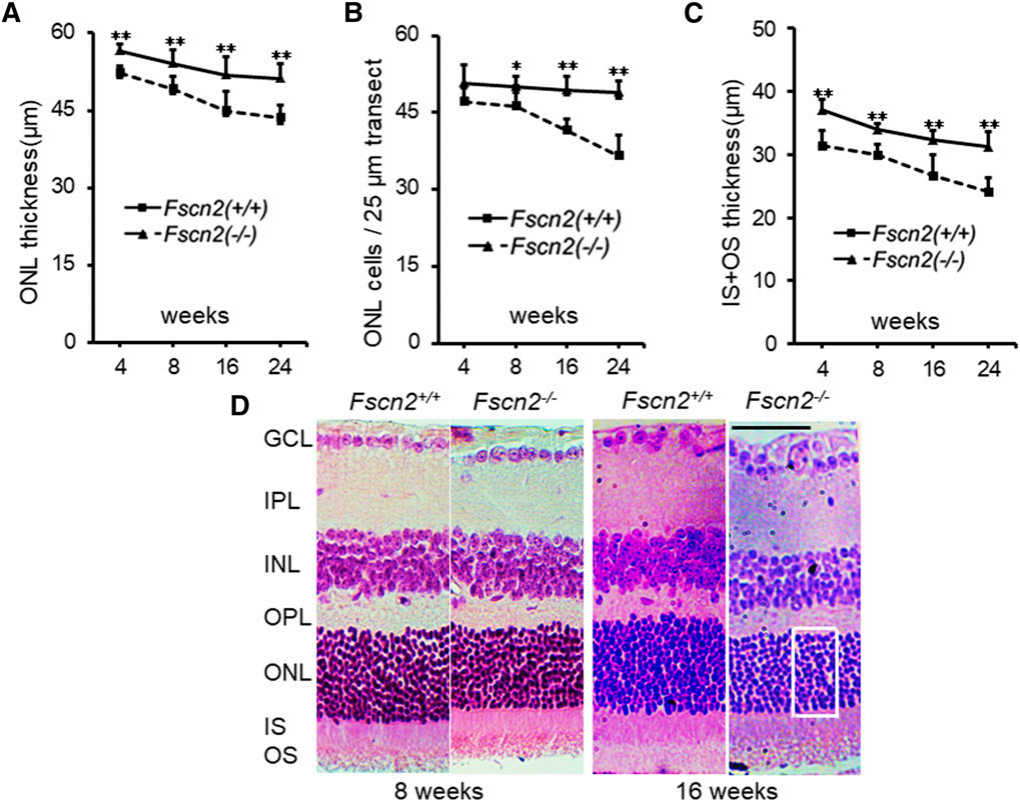
Histological analyses of retina. (A-C) Time course of changes in the central retinas: (A) thickness of the ONL, (B) nuclei in the ONL and (C) thickness of the IS and OS in the central retina. Cell density was assessed as the number of nuclei contained in a 25 µm-long section of the ONL. (n = 6 for each group at each time point). * *P* < 0.05; ** *P* < 0.01. (D) Typical Hematoxylin and Eosin stained sections of central retinas of 8 and 16 week-old *Fscn2^−/−^* mice. An area of the 25 µm width transect for measurement of ONL cells is indicated by a rectangle box. GCL, ganglion cell layer; IPL inner plexiform layer; INL, inner nuclear layer; OPL, outer plexiform layer; ONL, outer nuclear layer, IS, inner segments; OS, outer segments. Scale bars = 50 µm.

Dark-adapted (scotopic) flash ERGs were conducted on *Fscn2^−/−^* and *Fscn2^+/+^* mice at ages 4, 8, 12, and 24 weeks (Figure 8A and 8B). The amplitudes of the a-waves and b-waves in standard combined ERGs of *Fscn2^−/−^* mice were remarkably diminished compared with the levels for the wild-type mice of the same age. Typical images of standard combined ERGs in both mouse strains at ages 8 and 24 weeks re also presented (Figure 8C and 8D). The sections of the central retina were stained using hematoxylin and eosin (relative distance from the optic nerve head: 0.2 mm) in 4- to 24-week-old *Fscn2^−/−^* and *Fscn2^+/+^* mice. Generally, the *Fscn2^−/−^* mice showed thinner retinas at ages 8, 16, and 24 weeks; to be specific, the ONLs, and OSs and ISs were much thinner than those of the wild-type mice at these ages. Moreover, ONLs had fewer nuclei in *Fscn2^−/−^* mice than in the *Fscn2^+/+^* mice at ages 8, 16 and 24 weeks (Table 2 and Figure 9).

**Table 2:** The data for retinal measurement

Thickness of ONL (μm)				
	4 weeks	8 weeks	16 weeks	24 weeks
*Fscn-2^+/+^*	56.52 ± 1.37	54.00 ± 2.69	51.77 ± 3.58	51.20 ± 2.79
*Fscn-2^−/−^*	52.33 ± 1.28	49.13 ± 2.52	44.97 ± 3.81	43.53 ± 2.37
*P*	0.0003	0.0091	0.0097	0.0005

Thickness of IS+OS (μm)				
	4 weeks	8 weeks	16 weeks	24 weeks
*Fscn-2^+/+^*	37.17 ± 1.55	34.00 ± 0.89	32.35 ± 1.50	31.30 ± 2.40
*Fscn-2^−/−^*	31.48 ± 2.29	29.95 ± 1.71	26.72 ± 3.22	24.20 ± 2.12
*P*	0.0008	0.0011	0.0059	0.0003

Cell counts of ONL along a 25 µm width transect				
	4 weeks	8 weeks	16 weeks	24 weeks
*Fscn-2^+/+^*	50.80 ± 3.42	50.00 ± 2.00	49.40 ± 2.70	48.80 ± 2.28
*Fscn-2^−/−^*	47.00 ± 3.16	46.20 ± 3.09	41.40 ± 2.30	36.60 ± 3.97
*P*	0.1058	0.0112	0.0011	0.0008

## Discussion

In the present study, we first developed the *Fscn2* null-mutation (*Fscn2^−/−^)* mouse model using the C57BL/6J background. Earlier studies showed that the *Fscn2* gene was related to retinal pigmentosa in humans and mice(Wada *et al.* 2003; Wada *et al.* 2001; Yokokura *et al.* 2005). Later studies have shown a correlation between a mutation in *Fscn2* (p.R109H) and progressive hearing loss in DBA/2J mice(Shin *et al.* 2010). To fully understand the function of *Fscn2*, we established a mouse model with the *Fscn2* gene knocked out using TALEN(Sung *et al.* 2013). According to TALEN’s principles, multiple strains would be generated with different patterns of nucleotide deletion in the targeted gene. As a result, four F0 strains were developed with deletions in the *Fscn2* gene of 1, 4, 5, and 41 bp. All deletions were upstream of the coding sequence of the actin binding sites(Jansen *et al.* 2011; Lin *et al.* 2016), altered the open reading frame, and led to the null function of *Fscn2*. The strain carrying the 41-bp deletion in the *Fscn2* gene was further studied owing to its convenience in genotyping.

Next, we showed that the *Fscn2^−/−^* mice served as a new mouse model for age-related heating loss. The *Fscn2^−/−^* mice and the wild-type mice had nearly identical body shapes, coats, and weights. However, the *Fscn2^−/−^* mice demonstrated hearing loss with the following characteristics. (1) Hearing impairment in the *Fscn2^−/−^* mice occurred at about 3 weeks of age, which indicated early-onset hearing loss. (2) The hearing impairment was more sensitive to pure tone stimuli at high frequencies (such as 16 and 32 kHz) than to those at a low frequency (8 kHz). This phenomenon corresponded to the loss of OHCs (starting in the basal turns and then spreading to the apical turns) in several mouse models of age-related hearing loss(Han *et al.* 2012; Zheng *et al.* 2009). (3) The hearing impairment was progressive in the one-year period of observation. The ABR thresholds in the *Fscn2^−/−^* mice rose, starting at age 3 weeks and reaching about 100 dB SPL by age 24 weeks. The mice became deaf afterward. The chronic progressive hearing loss in *Fscn2^−/−^* mice thus provides a time window for further mechanistic investigation and drug intervention.

Degeneration of hair cells is responsible for hearing loss in *Fscn2^−/−^* mice. A pathological study indicated that the *Fscn2^−/−^* mice suffered from progressive degeneration of OHCs, starting in the cochlear basal turns at age 6 weeks. Observation of the stereocilia using scanning electron microscopy further revealed impairment of the hair-cell bundles at age 3 weeks. It was reported that, in the embryonic period, increment of Fascin2 expression was in proportion with the growth of the stereocilia(Avenarius *et al.* 2014). Although the stereocilia in *Fscn2^−/−^* mice existed in the cochleae after birth, they had defects. For example, at age 3 weeks, the stereocilia were already irregular in shape. They then became shorter and less stiff. Impairment of the stereocilia became severe with age, as the bundles did not have enough strength to withstand the repeated sound stimuli. Moreover, the decrement in DPOAE amplitudes in the age range of 3 to 6 weeks was a reflex caused by the functional impairment in the OHCs. Therefore, degeneration of the hair cells or the stereocilia is the primary cause of hearing loss in *Fscn2^−/−^* mice.

Lastly, we showed that the *Fscn2^−/−^* mice served as a new mouse model for retinal degeneration. It has been reported that 208delG in FSCN2 correlated with autosomal-dominant retinitis pigmentosa and autosomal-dominant macular degeneration in the Japanese population(Wada *et al.* 2003; Wada *et al.* 2001). Therefore, in this study, we tested the standard combined ERG for the *Fscn2^−/−^* mice. The b-wave and a-wave amplitudes of the standard combined ERG of *Fscn2^−/−^* mice were remarkably lower than those of the wild-type mice at ages 4, 8, 12, and 24 weeks. Histochemical observation revealed that the *Fscn2^−/−^* mice had thinner retinas, especially for OSs, with fewer nuclei in ONLs, than did the wild-type mice at ages 8,16 and 24 weeks. The results coincided with those of previous studies(Yokokura *et al.* 2005). However, the immunohistochemical results indicated that Fscn2 was expressed in the OSs, IPLs, and OPLs of mouse retinas. As Fscn2 is an actin binding protein, it is reasonable that the protein exists in multiple layers of the retinas. These results indicate that pathological degeneration in the retinas causes functional impairment or abnormal ERG in *Fscn2^−/−^* mice.

It souled be mentioned that hearing loss in *Fscn2^−/−^* mice is not as severe as it is in DBA/2J mice. This may be because, in addition to the mutations in *cdh23* (*ahl*) and *Fscn2* (*ahl8*), the quantitative trait locus on chromosome 5 may also be responsible for severe hearing loss in DBA/2J mice(Johnson *et al.* 2015; Suzuki *et al.* 2015).

## Conclusion

In summary, the knockout of the Fscn2 gene in C57BL/6J mice causes progressive hearing loss and degeneration of hair cells and retinas. This model will aid in functional studies of Fscn2 to increase understanding of the etiology of FSCN2-associated disorders in humans.
